# Impact of easing COVID-19 safety measures on trauma computed tomography imaging volumes

**DOI:** 10.1007/s10140-022-02096-4

**Published:** 2022-10-29

**Authors:** Sriram Rao, Justin Glavis-Bloom, David Kakish, Karen Tran-Harding, Daniel S. Chow, Michael Nguyentat, Eric O. Yeates, Jeffry Nahmias, Roozbeh Houshyar

**Affiliations:** 1 Department of Radiological Sciences, University of California Irvine, Orange, CA USA; 2 Department of Surgery, University of California Irvine, Orange, CA USA

**Keywords:** COVID-19, Coronavirus, ER, Trauma, Healthcare utilization

## Abstract

**Purpose:**

The coronavirus disease 2019 (COVID-19) pandemic has led to substantial disruptions in healthcare staffing and operations. Stay-at-home (SAH) orders and limitations in social gathering implemented in spring 2020 were followed by initial decreases in healthcare and imaging utilization. This study aims to evaluate the impact of subsequent easing of SAH on trauma volumes, demand for, and turnaround times for trauma computed tomography (CT) exams, hypothesizing that after initial decreases, trauma volumes have increased as COVID safety measures have been reduced.

**Methods:**

Patient characteristics, CT imaging volumes, and turnaround time were analyzed for all adult activated emergency department trauma patients requiring CT imaging at a single Level-I trauma center (1/2018–2/2022) located in the sixth most populous county in the USA. Based on COVID safety measures in place in the state of California, three time periods were compared: baseline (PRE, 1/1/2018–3/19/2020), COVID safety measures (COVID, 3/20/2020–1/25/2021), and POST (1/26/2021–2/28/2022).

**Results:**

There were 16,984 trauma patients across the study (PRE = 8289, COVID = 3139, POST = 5556). The average daily trauma patient volumes increased significantly in the POST period compared to the PRE and COVID periods (13.9 vs. 10.3 vs. 10.1, *p* < 0.001), with increases in both blunt (*p* < 0.001) and penetrating (*p* = 0.002) trauma. The average daily number of trauma CT examinations performed increased significantly in the POST period compared to the PRE and COVID periods (56.7 vs. 48.3 vs. 47.6, *p* < 0.001), with significant increases in average turnaround time (47 min vs. 31 and 37, *p* < 0.001).

**Conclusion:**

After initial decreases in trauma radiology volumes following stay-at-home orders, subsequent easing of safety measures has coincided with increases in trauma imaging volumes above pre-pandemic levels and longer exam turnaround times.

**Supplementary Information:**

The online version contains supplementary material available at 10.1007/s10140-022-02096-4.

## Introduction 

The coronavirus disease 2019 (COVID-19) pandemic has caused substantial disruptions in healthcare staffing and operations throughout multiple surges in cases of COVID-19. Following the first major wave of infections in spring 2020, limitations on public gatherings were instituted across the USA [1]. On March 19, 2020, the California governor and Department of Public Health issued a shelter-in-place order for all individuals to “stay home or at their place of residence, except as needed to maintain continuity of operation of the federal critical infrastructure sectors” [2]. We previously reported that in the 2-week period following this order, average daily trauma-related emergency department imaging volume decreased by 30% at several major academic medical centers in California [3]. Similar decreases in emergency department and trauma volumes across the USA have been reported initially following institution of COVID-19 safety measures [4–8].

Throughout the remainder of 2020, initial stay-at-home (SAH) orders were replaced with various mitigation measures, including regional SAH orders, limitations on business reopening, and nighttime curfews. After transiently decreasing, multiple institutions reported overall trauma volumes quickly returned to baseline levels, with increases in penetrating trauma and firearm injuries, including special populations such as children [8–15]. Similarly, after initially decreasing, the number of motor vehicle collisions and fatalities increased nationally in 2020, with increases in risky behaviors including speeding, failure to wear seatbelts, and driving under the influence of alcohol or other drugs [16, 17].

On January 25, 2021, as vaccine availability increased and the “delta wave” of the pandemic began to recede, California’s Department of Public Health lifted the regional SAH orders that had replaced the statewide shelter-in-place order [18]. Subsequently on June 15, 2021, California “fully reopened,” eliminating most remaining mitigation efforts excepting large events and healthcare settings [19]. However, there are a paucity of studies examining how trauma volumes, patient characteristics, and computed tomography (CT) imaging practices changed as COVID-19 safety measures were lifted.

Therefore, this study aimed to review how easing of COVID-19-related public health measures corresponded with demand for Emergency Department (ED) trauma radiology services. We hypothesized that after initial decreases, trauma volumes have increased as COVID safety measures have been reduced. Understanding trends in trauma volumes is essential to resource allocation planning, as trauma patients frequently require numerous CT examinations with rapid turnaround time, which potentially delays evaluation of other critically ill patients awaiting radiologic examination.

## Methods

This study was approved by our Institutional Review Board, and a waiver of consent was granted. Next, a database of radiology reports was queried (1/1/2018–2/28/2022) for all activated ED trauma patient CT radiology reports using mPower Clinical Analytics (Nuance, Burlington, MA). This database was correlated with a separate registry maintained by our trauma surgery department of every adult trauma patient presenting to our medical center, a Level-1 trauma center in the sixth most-populous county in the USA. All trauma patients who required any CT evaluation were included, and no patients were excluded.

The mean daily volumes of trauma CT radiologic examinations and trauma patient characteristics were assessed using the following time periods: baseline period (PRE, from January 1, 2018, to March 19, 2020, the date of California’s statewide shelter-in-place order), COVID safety measures period (COVID, from March 19, 2020, to January 25, 2021, the date of lifting of regional stay-at-home orders), and POST period from January 25, 2021, until February 28, 2022. Daily averages were used to normalize for different time period lengths.

Traumas were classified as blunt or penetrating. Blunt traumas were further subclassified into assault, fall, auto versus pedestrian (AVP), motorcycle collision (MCC), and motor vehicle collision (MVC). Penetrating traumas were subclassified into gunshot wounds (GSW) and stab wounds (SW).

Trauma severity was analyzed using the Abbreviated Injury Scale (AIS) and the Injury Severity Score (ISS). The AIS is a 6-point coding system of injury severity ranging from minor to severe trauma, calculated separately for the head, face, chest, abdomen/pelvis, extremities, and external/other, with a score of 3 corresponding to severe injury. The ISS is calculated as the sum of the squares of the three highest AIS severity codes and ranges from 1 to 75, with a score greater than 15 corresponding to major trauma [20].

Additionally, the average number of CT examinations performed per patient were analyzed for each period. The average turnaround time (TAT) was calculated as the difference between the time a CT was ordered and was marked as completed by the CT technologist in the radiology information system.

Descriptive and inferential statistics were performed using the R statistical software (v4.1.0, R Foundation for Statistical Computing, Vienna, Austria). Categorical variables were compared across the three study periods with chi-squared (*χ*^2^) distribution and Fisher exact tests. Continuous variables were compared across the three periods using analysis of variance (ANOVA), and between each period using post hoc Tukey testing with correction for multiple tests. Results with *p* < 0.05 were considered significant.

## Results

A total of 16,984 patients were recorded during the entire study period, 8289 during the PRE period, 3139 during the COVID period, and 5556 during the POST period. Patient characteristics of each study period are outlined in Table 1.

**Table 1:** Trauma patient demographics and injury severity

	PRE (*N* = 8289)	COVID (*N* = 3139)	POST (*N* = 5556)	*p*-value*
Demographics				
Age, mean years ± SD	48.6 ± 24.1	49.7 ± 23.2	49.8 ± 24.3	0.007
Sex, *n* (%)				
Male	5,095 (61%)	2,079 (66%)	3,419 (62%)	
Female	3,194 (39%)	1,060 (34%)	2,137 (38%)	
Mean daily patients	10.3	10.1	13.9	< 0.001
Blunt trauma	9.2	8.7	12.1	< 0.001
Assault	0.5	0.6	0.7	0.002
Auto versus pedestrian	1.1	0.9	1.1	0.119
Fall	2.9	3.2	4.3	< 0.001
Motorcycle collision	0.8	0.8	0.9	0.657
Motor vehicle collision	3.8	3.2	5.0	< 0.001
Penetrating trauma	0.6	0.8	0.9	0.002
Gunshot wound	0.2	0.3	0.3	0.251
Stab wound	0.4	0.5	0.6	0.005
Other	0.5	0.6	0.9	< 0.001
Injury severity				
AIS ≥ 3 (*n* (%))				
Head	1,235 (16%)	434 (15%)	626 (12%)	< 0.001
Face	38 (0%)	17 (1%)	11 (0%)	0.010
Chest	1,123 (14%)	413 (14%)	626 (12%)	0.002
Abdomen	267 (3%)	96 (3%)	201 (4%)	0.224
Extremity	590 (7%)	238 (8%)	385 (7%)	0.580
External/Other	12 (0%)	7 (0%)	23 (0%)	0.009
ISS > 15 (n (%))	1,116 (13%)	422 (14%)	670 (12%)	0.140

*SD* standard deviation, *AIS* Abbreviated Injury Scale, *ISS* injury severity score

^*^p-values calculated with analysis of variance (ANOVA) for continuous variables and Fisher exact tests for categorical variables

### Demographics, mechanisms of injury, and injury severity

Mean age and sex were similar between the three cohorts. However, the average daily trauma patient volumes increased significantly in the POST period compared to the PRE and COVID periods (13.9 vs. 10.3 vs. 10.1, *p* < 0.001).

There were also differences in mechanisms of injury between the study periods. A higher incidence of blunt traumas occurred during the POST period compared to PRE (12.1 mean daily traumas vs. 9.2, *p* < 0.001), with increases in assaults (0.7 vs. 0.5, *p* = 0.003), motor vehicle collisions (0.9 vs 0.8, *p* < 0.001), and falls (4.3 vs. 2.9, *p* < 0.001); in each of these categories, there were also statistically significant increases in the POST period compared to the COVID period (Supplemental Table 1). In addition, there were significant increases in penetrating traumas in the POST period compared to PRE (0.9 vs. 0.6, *p* = 0.001), with increases in stab wounds (0.6 vs. 0.4, *p* = 0.004). In regard to injury severity, there was a decreased proportion of patients with severe injuries (AIS ≥ 3) during the POST period compared to the PRE cohort for the head (12% vs. 16%, *p* < 0.001), face (0% vs. 0%, *p* = 0.038), and chest (12% vs. 14%, *p* = 0.01) and decreased injuries involving the head and face in the POST cohort compared to the COVID cohort (Supplemental Table 1). However, there was a similar proportion of patients with overall major injury severity (ISS > 15) across all cohorts.

### CT imaging and turnaround times

The average daily number of CT examinations performed increased significantly in the POST period compared to the PRE and COVID periods (56.7 vs. 48.3 vs. 47.6, both *p* < 0.001). There was no difference in the average daily number of CT examinations performed between the COVID and PRE periods. Despite the overall increase in CT examinations in the POST period, interestingly, the mean number of CT examinations performed per patient decreased in the POST period (4.4) compared to the PRE (4.9) and COVID (5.1) periods (both *p* < 0.001). There was also a significant difference in the mean number of CT examinations performed per patient between the COVID and PRE periods (*p* = 0.029) (Table 2).

**Table 2:** Trauma CT imaging examination characteristics

	PRE	COVID	POST	*p*-value*
Total examinations	40,835	15,804	23,514	
By division (*n* (%))				0.010
Neuro	19,723 (48%)	7665 (49%)	12,157 (52%)	
Chest	7187 (18%)	2750 (17%)	3833 (16%)	
Body	7105 (17%)	2734 (17%)	3809 (16%)	
Musculoskeletal	6819 (17%)	2655 (17%)	3715 (16%)	
Mean daily examinations	48.3	47.6	56.7	< 0.001
Mean exams per patient	4.9 ± 2.1	5.1 ± 2.2	4.4 ± 1.9	< 0.001
Turnaround time (minutes)	31	37	47	< 0.001

^*^p-values calculated with analysis of variance (ANOVA) for continuous variables and chi-squared (*χ*^2^) distribution for categorical variables

There was an increased mean turnaround time in the POST period (47 min) compared to the PRE (31 min) and COVID (37 min) periods, and also a significant difference between the COVID and PRE periods (all three *p* < 0.001).

## Discussion

In this retrospective study, there was an approximately 35% increase in trauma patients and a 17% increase in daily number of trauma CT examinations during the POST period compared to the PRE period, following statewide easing of COVID safety measures. However, there was a decrease in the mean number of CT examinations performed per patient and an increase in the turnaround time during the POST period when compared to the PRE and COVID periods.

Multiple prior publications have reported transient decreases in ED and trauma volumes after institution of COVID-19 safety measures, followed by increases in penetrating trauma and gunshot injuries [8–13]. However, there is a paucity of studies examining long-term changes in trauma cases and trauma CT volumes during the later stages of the pandemic, as safety measures have been substantially decreased and essentially eliminated in many settings.

Turnaround time is an important metric for ED CT imaging as it reflects access to diagnosis and thereby treatment in a potentially critically ill cohort. Corresponding with the increase in trauma patients and CT examinations, this study demonstrated a 10-min or more increase in examination turnaround time compared to the prior cohorts. Turnaround times are impacted by multiple factors, including staffing and equipment utilization, and may reflect a need for additional resources, for example, in transport and technologist personnel or equipment [21, 22]. While this particular study lacks granular data to identify which factors contributed to the observed increase in turnaround time, one potential explanation may be as simple as the increase in trauma volume. Although we are not aware of any adverse events specifically related to delayed CT examination, timely completion of trauma CT examinations is an essential component of appropriate triage and optimal care [23–25]. In addition, imaging of trauma patients can delay evaluation of other ED patients awaiting CT examinations, as trauma patients essentially “skip the line” of patients waiting to be imaged. As trauma cases increased in the POST period, there may have been delays in CT turnaround time for non-trauma emergency department patients, which merits further research.

Reports of increased penetrating trauma have been rampant following the wake of the COVID-19 pandemic [26, 27]. In the present analysis, we found an increased rate of assaults (44% increase) and overall penetrating trauma (also a 44% increase) in the POST period compared to the PRE period, which coincide with increased firearms sales associated with the COVID-19 pandemic [28–31]. In addition, this study found a 32% increase in motor vehicle collisions, which may be partially explained by other studies finding an increase in risky driving behaviors including driving under the influence of alcohol, speeding, and failure to wear seatbelts in 2020, the most recent year for which data is available [32, 33]. While this data is concerning, further multicenter studies are needed to confirm the generalizability of these trends and attempt to elucidate if there are any opportunities for primary prevention to curtail these concerning findings.

This study has inherent limitations by virtue of its retrospective single-center design. In addition, as previously mentioned due to the single-center design, this study may lack generalizability. Also, although COVID safety measures during the study period represent an important societal event, we did not control for additional variables which may have had an impact, including other societal factors, seasonality, or longer-term trends in trauma volumes and mechanisms.

## Conclusion

The COVID-19 pandemic has had substantial impacts on healthcare, social determinants of health, and society at large. This study spanning 3 periods surrounding the COVID-19 pandemic found significant increases in overall trauma volumes and turnaround times for CT imaging during the most recent POST phase of the COVID-19 pandemic, following substantial decreases in COVID safety measures. These findings suggest that additional resources, innovative approaches, and further research are necessary to effectively provide emergent imaging for an increasing trauma population.

## Supplementary Information

Below is the link to the electronic supplementary material.

Supplementary file1 (DOCX 14 KB)

Supplementary file2 (DOCX 13 KB)
