# Supplementary material for: Impact of easing COVID-19 safety measures on trauma computed tomography imaging volumes
Source: Emerg Radiol. 2022 Oct 29;30(1):27–32. doi: 10.1007/s10140-022-02096-4 (PMC9616698; doi:10.1007/s10140-022-02096-4)
Supplement: Supplementary file 1 — Supplementary file1 (DOCX 14 KB) [file 10140_2022_2096_MOESM1_ESM.docx]

**Supplemental Table 1. Trauma Patient Demographics and Injury Severity, Pairwise Tests**

|  | **PRE vs. COVID** | **PRE vs. POST** | **COVID vs. POST** |
| --- | --- | --- | --- |
| **Demographics** |  |  |  |
| **Age** | 0.077 | 0.010 | 0.972 |
| **Mean Daily Patients** | 0.079 | <0.001 | <0.001 |
| Blunt Trauma | 0.119 | <0.001 | <0.001 |
| Assault | 0.994 | 0.003 | 0.014 |
| Auto Versus Pedestrian | 0.129 | 0.994 | 0.166 |
| Fall | 0.124 | <0.001 | <0.001 |
| Motorcycle Collision | 0.990 | 0.687 | 0.717 |
| Motor Vehicle Collision | 0.003 | <0.001 | <0.001 |
| Penetrating Trauma | 0.693 | 0.001 | 0.064 |
| Gunshot Wound | 0.585 | 0.233 | 0.888 |
| Stab Wound | 0.856 | 0.004 | 0.081 |
| Other | 0.089 | <0.001 | 0.158 |
| **Injury Severity** |  |  |  |
| **AIS >= 3** (n (%)) |  |  |  |
| Head | 0.510 | <0.001 | 0.003 |
| Face | 0.999 | 0.038 | 0.030 |
| Chest | 0.999 | 0.010 | 0.057 |
| Abdomen | 0.999 | 0.447 | 0.422 |
| Extremity | 0.999 | 0.999 | 0.999 |
| External/Other | 0.999 | 0.008 | 0.548 |
| **ISS > 15** (n (%)) | 0.999 | 0.232 | 0.318 |

* p-values calculated with post-hoc Tukey tests for continuous variables and Fisher exact tests for categorical variables; SD = standard deviation; AIS = Abbreviated Injury Scale; ISS = Injury Severity Score
