# Supplementary material for: Impact of easing COVID-19 safety measures on trauma computed tomography imaging volumes
Source: Emerg Radiol. 2022 Oct 29;30(1):27–32. doi: 10.1007/s10140-022-02096-4 (PMC9616698; doi:10.1007/s10140-022-02096-4)
Supplement: Supplementary file 2 — Supplementary file2 (DOCX 13 KB) [file 10140_2022_2096_MOESM2_ESM.docx]

**Supplemental Table 2. Trauma CT Imaging Examination Characteristics, Paired Comparisons**

|  | **PRE vs. COVID** | **PRE vs. POST** | **COVID vs. POST** |
| --- | --- | --- | --- |
| Total Examinations | 0.398 | 0.004 | 0.018 |
| Mean Daily Examinations | 0.991 | <0.001 | <0.001 |
| Mean Exams per Patient | 0.029 | <0.001 | <0.001 |
| Turnaround Time (minutes) | <0.001 | <0.001 | <0.001 |

* p-values calculated with post-hoc Tukey tests for continuous variables and chi-squared (χ2) distribution for categorical variables
